# Increase of Suicide in France

**Published:** 1848-11

**Authors:** 


*Increase of Suicide in France.*—According to some statistical tables for the year 1846, recently published in France, it appears that suicide has been for some years progressively on the increase in that country; and as we infer from the report to an amount greater than would be indicated by the increase of population. The number of suicides amounted in

| | | |
|---|---|---|
| 1841 | to - - - - - | 2814 |
| 1842 | to - - - - - | 2886 |
| 1845 | to - - - - - | 3084 |
| 1846 | to - - - - - | 3102 |

Amongst the suicides in 1846, were 27 children from 10 to 15 years of age, 139 between 16 and 21 years, 443 from 21 to 30 years, 1,214 from 30 to 50 years, 513 from 50 to 60 years, 403 from 60 to 70 years, 209 from 70 to 80 years, and 51 above 80 years. Suicides are more frequent in spring and summer than in winter and autumn. The months of June, July, and August, produced 940. Those of March, April, and May, 904. Those of September, October, and November, 654; and those of December, January and February, 604. Strangulation and suspension were the means most frequently employed by the suicides of 1846 to terminate their existence: 1,077 had recourse to it; 1,036 drowned themselves; 222 suffocated themselves with charcoal, and 429 used fire-arms. Amongst the suicides recorded in the year 1846, more than a quarter, viz., 888, were caused by insanity. The other cases arose from various causes. Physical sufferings and domestic unhappiness, pecuniary embarrassment, and the fear of distress, are the most frequent.

It further appears that the number of accidental deaths during the year 1846, amounted to 7,558: of which 3,861 were caused by drowning, 624 were crushed to death by carriages, and 45 were caused by railroad accidents.—*Medical Gazette.*

